# Supplementary material for: The mycomembrane proteins PorH and ProtX are inserted at polar growth zones and linked to the cell wall
Source: bioRxiv. 2025 Oct 14:2025.10.14.682376. Preprint. [Version 1] doi: 10.1101/2025.10.14.682376 (PMC12632848; doi:10.1101/2025.10.14.682376)
Supplement: Supplement 1 [file media-1.pdf]

SUPPLEMENTAL MATERIAL

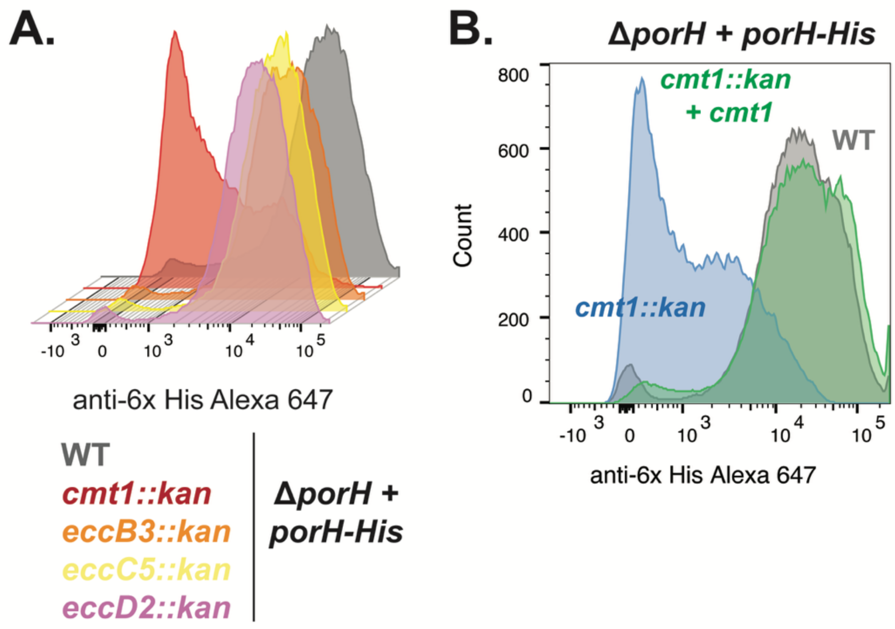

Figure S1: Surface assembly of PorH requires Cmt1 but not the type VII secretion system. Flow cytometry detection of PorH-His with anti-6x His antibody labeled with Alexa647. The PorH-His construct was expressed in the indicated genetic backgrounds. A representative replicate is displayed as a histogram. In all strains, the native copy of *porH* is deleted and PorH-His is constitutively expressed from a replicating plasmid (1, 2). **(A)** WT or cells in which genes encoding predicted type VII secretion system components were deleted. **(B)** Wild-type (WT) cells, cells lacking *cmt1*, or cells lacking *cmt1* with ectopic *cmt1* complementation. Complementation of *cmt1::kan* was performed by integrating an ectopic *cmt1* expression from a chromosomally integrated construct and inducing expression with theophylline (3, 4).

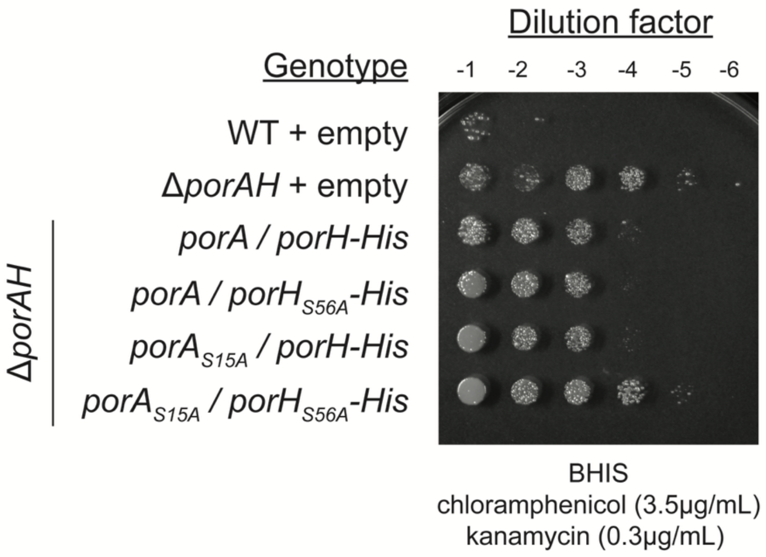

Figure S2: Preventing *O*-mycoloylation of PorH or PorA is required for PorAH function. Ten-fold serial dilutions of the indicated strains were spotted onto BHIS media containing 0.3μg/mL kanamycin. The *porA/porH-His* alleles were constitutively expressed from a replicating vector in a background lacking the native *porAH* locus and “+ empty” indicates that the strain carries an empty vector control. Chloramphenicol was included for plasmid maintenance.

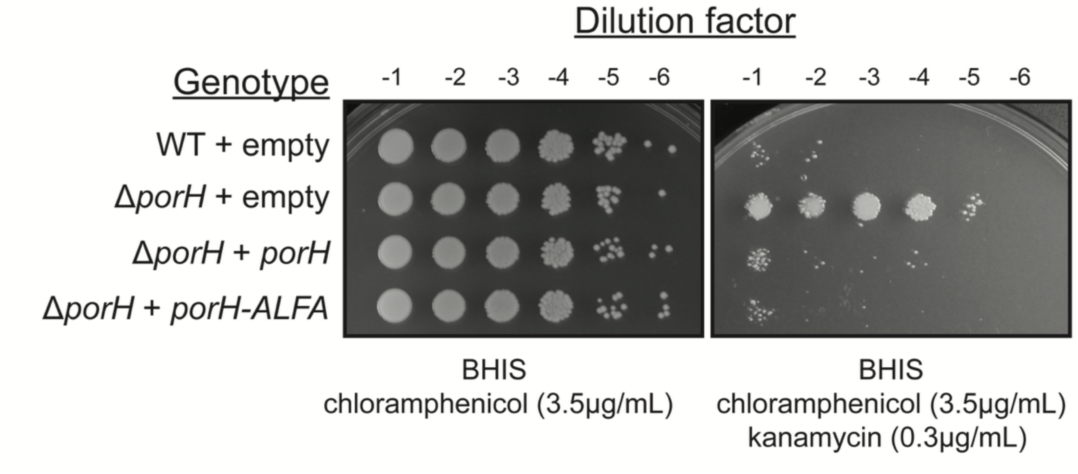

Figure S3: Ectopic PorH-ALFA complements *porH* null kanamycin resistance. Ten-fold serial dilutions of the indicated stains were spotted onto BHIS media or BHIS media containing 0.3μg/mL kanamycin. Strains carry either an empty vector ("+ empty") or the indicated complementation vector from which PorH or PorH-ALFA was constitutively expressed. Chloramphenicol was included for plasmid maintenance.

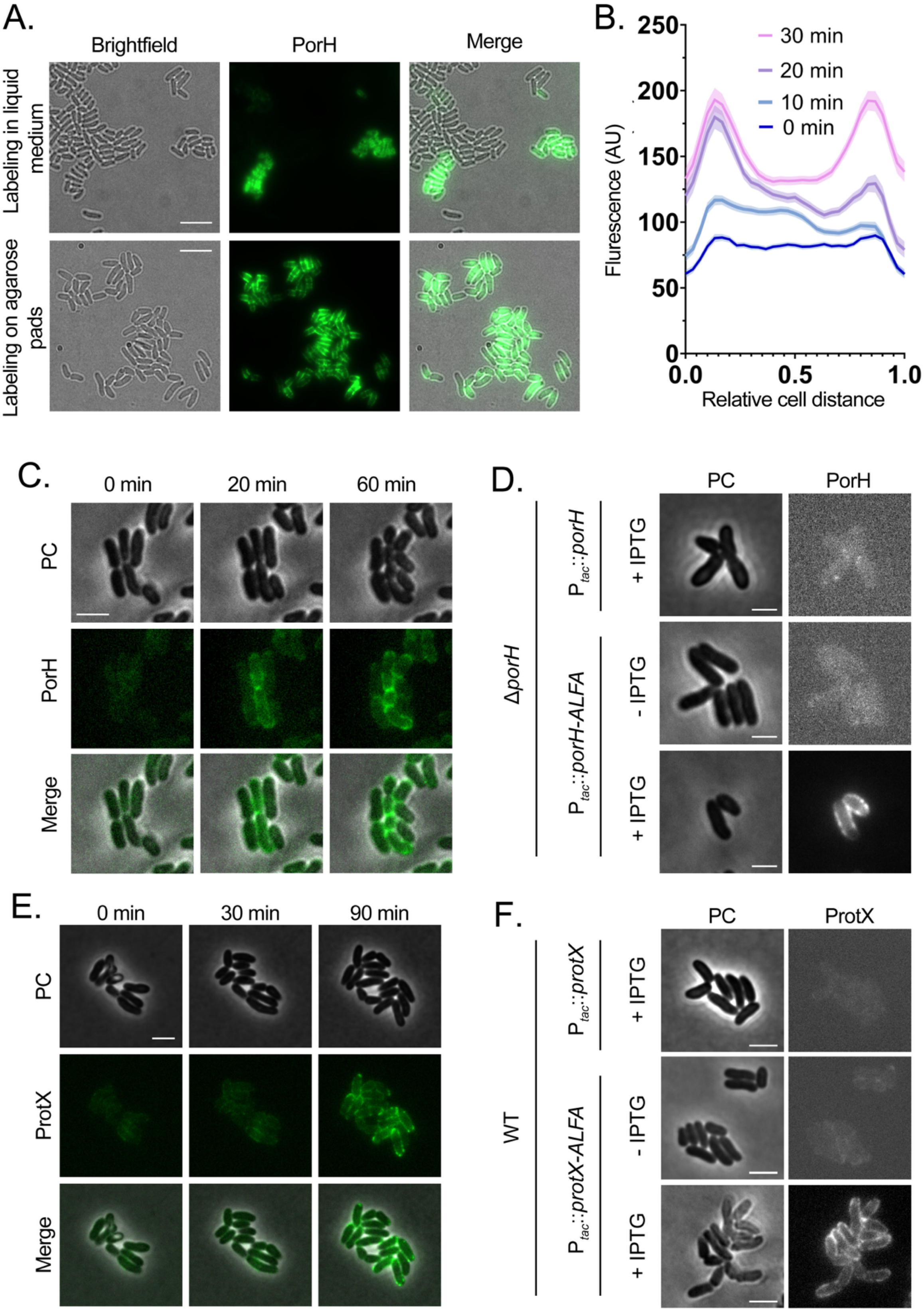

A.
Brightfield
PorH
Merge
Labeling in liquid medium
Labeling on agarose pads
B.
250
200
150
100
50
0
Flurescence (AU)
30 min
20 min
10 min
0 min
0.0
0.5
1.0
Relative cell distance
C.
0 min
20 min
60 min
PC
PorH
Merge
D.
PC
PorH
ΔporH
P tac::porH
+ IPTG
P tac::porH-ALFA
- IPTG
+ IPTG
E.
0 min
30 min
90 min
PC
ProtX
Merge
F.
PC
ProtX
WT
P tac::protX
+ IPTG
P tac::protX-ALFA
- IPTG
+ IPTG

Figure S4: Specific detection of PorH-ALFA and ProtX-ALFA using the anti-ALFA nanobody. **(A)** *Cglu* strain DB001 (Δ*porH*, P_{tac}::*porH-ALFA*) was induced with 500 µM IPTG for 2h in liquid (top) or on solid (bottom) BHI medium supplemented with 3.5µg/ml chloramphenicol. Cells were stained with 12.5 nM Atto488-NB and imaged by brightfield and fluorescence channel. Scale bars = 6 µm. **(B)** Quantification of Atto488-NB labeled ProtX fluorescence intensity as a function of normalized cell length at indicated time points following IPTG induction. Cells were oriented based on highest ProtX intensity (= Pole 0). Representative graph from one biological replicate (n ≥ 316 cells), shaded area indicates ± 95% confidence interval **(C)** Representative images of cells (strain H1616) expressing PorH-ALFA induced with 50 µM IPTG and stained with 12.5 nM Atto488-NB at indicated timepoints. PC = phase contrast, scale bar = 3 µm. **(D)** Cells expressing untagged PorH or PorH-ALFA with no IPTG or 500 µM IPTG induction, where indicated. Scale bars = 2 µm. **(E)** Representative images of cells (strain DB046) expressing ProtX-ALFA induced with 50 µM IPTG and stained with 12.5 nM Atto488-NB at indicated timepoints. Scale bar = 3µm. **(F)** Cells expressing untagged ProtX or ProtX-ALFA imaged with no IPTG or with 500 µM IPTG induction, where indicated. Scale bars = 3 µm.

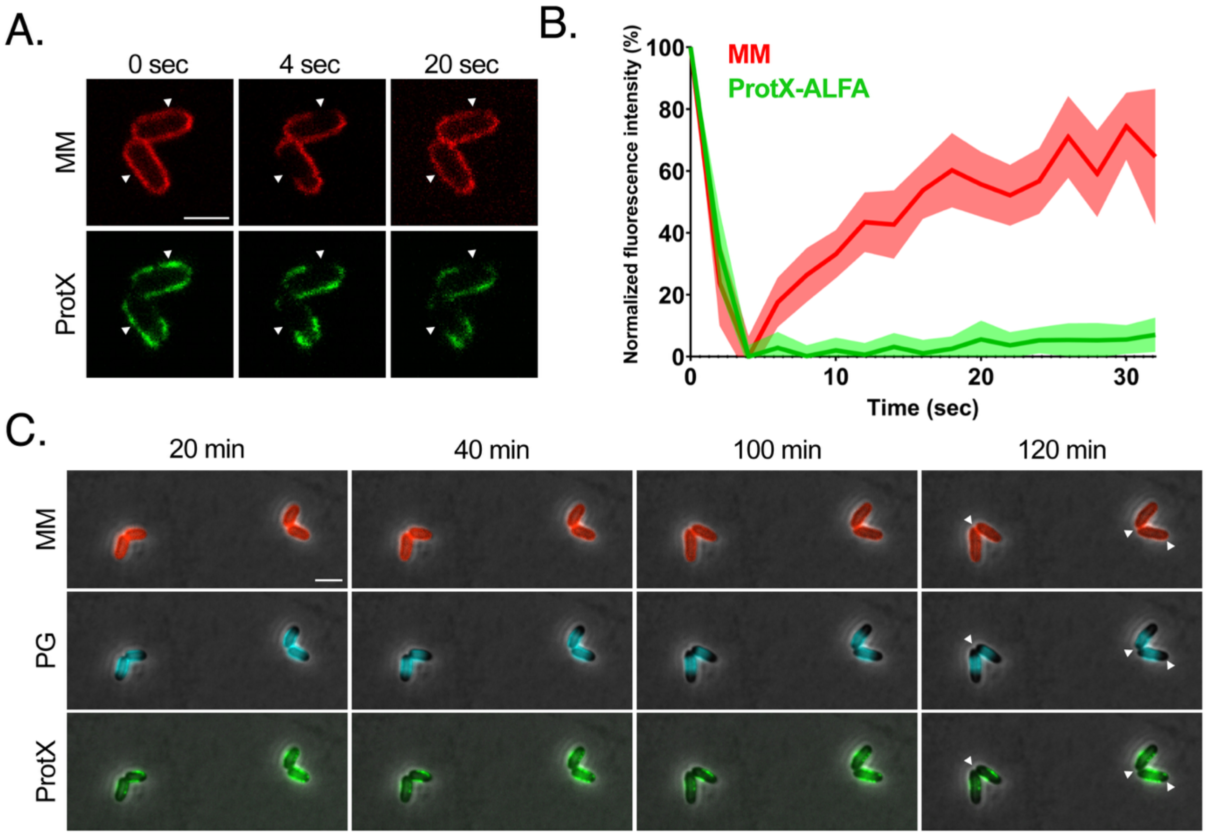

Figure S5. ProtX remains non-diffusive upon its incorporation into the MM. (A) Representative images of photobleached cells (strain DB42) labeled with 30 μM 6-TMR-tre (MM = mycomembrane) and 12.5 nM Atto488-NB labeling ProtX-ALFA. Arrowheads indicate bleached regions. Scale bar = 2μm. **(B)** FRAP recovery curve for MM and ProtX. Line indicates mean, shaded area indicates 95% confidence interval, n = 77. **(C)** Fluorescence dilution assay for ProtX-ALFA. Cells (strain DB42) were prelabeled with same dyes as in **(A)** and imaged over 4h with 20min acquisition frame rate. Arrowheads indicate regions of new cell envelope insertion at the pole. Scale bar = 3 μm.

A.

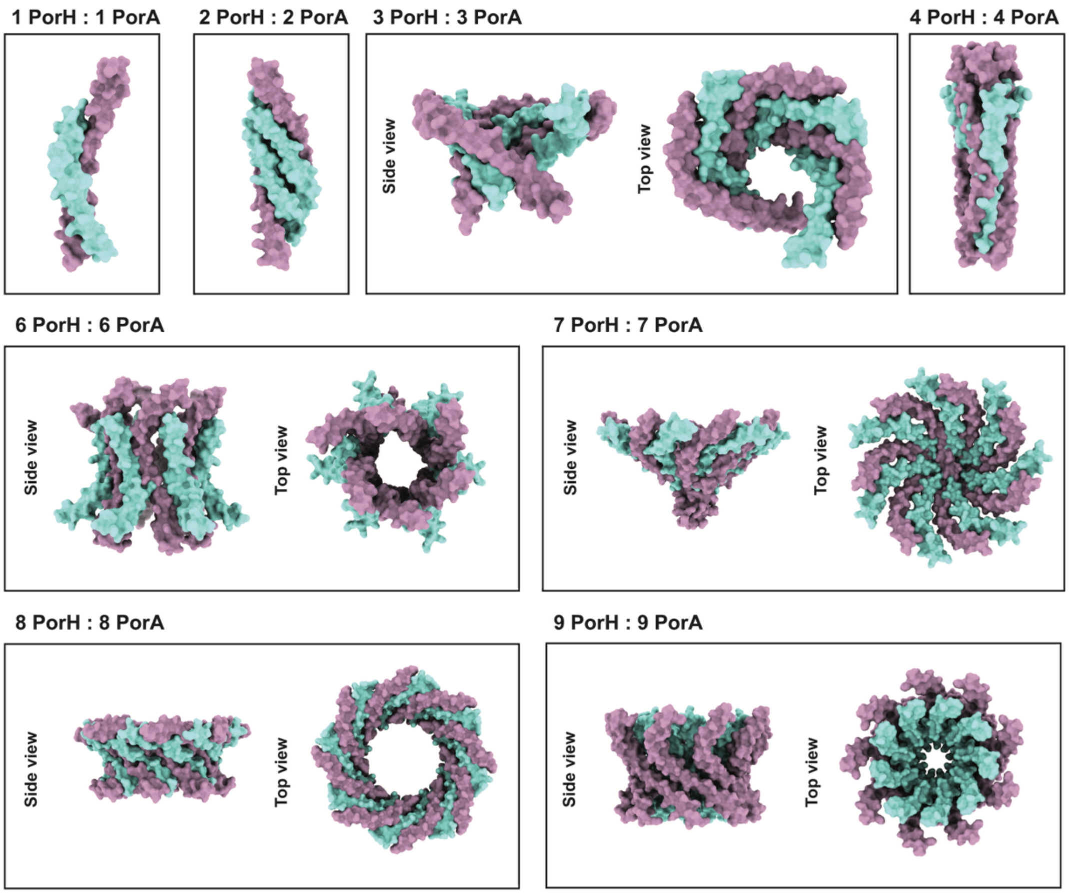

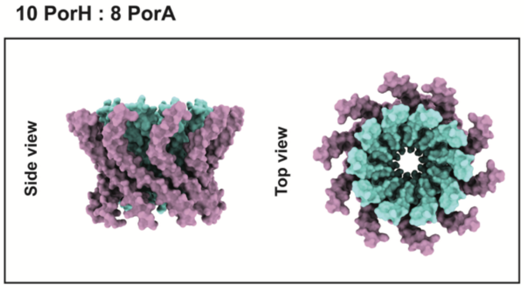

B.

Hetero-oligomer	ipTM	pTM	pLDDT
1 PorH : 1 PorA	0.216	0.42	66
2 PorH : 2 PorA	0.32	0.45	59.7
3 PorH : 3 PorA	0.86	0.87	95.7
4 PorH : 4 PorA	0.72	0.74	51.7
5 PorH : 5 PorA	0.883	0.889	94.5
6 PorH : 6 PorA	0.118	0.17	33.3
7 PorH : 7 PorA	0.126	0.172	34.5
8 PorH : 8 PorA	0.23	0.27	54.3
9 PorH : 9 PorA	0.174	0.205	34.7
10 PorH : 10 PorA	0.184	0.211	35.9

Figure S6: Predicted PorAH hetero-oligomeric structures. (A) Structural predictions of different hetero-oligomers of PorA and PorH (PorA in pink and PorH in cyan) predicted by AlphaFold2 (5). Only the Rank 1 prediction of each input is displayed (B) AlphaFold2 confidence measurements of the structures shown in **(A)** and Figure 6.

Supplementary Movie Legend

Movie S1: Live-cell imaging of PorH surface assembly. Cells (H3922, Δ*porH* P_{tac}*::porH-ALFA*) were spotted on a 2% agarose pad containing 500 µM IPTG and 12.5 nM diluted Atto488-NB. PorH-ALFA surface assembly was observed over a 2 hr observation period with a 2:30 min acquisition frame rate. Three examples are shown, PC = phase contrast, scale bar = 2 µm. Movie is rendered at 10 frames per seconds.

Movie S2: Live-cell imaging of ProtX incorporation. Cells (DB042*,* P_{tac}*::protX-ALFA*) were spotted on a 2% agarose pad containing 500 µM IPTG and 12.5 nM diluted Atto488-NB. ProtX-ALFA surface assembly was observed over a 2 hr observation period following a 2:30 min acquisition frame rate. Three examples are shown, PC = phase contrast, scale bar = 2µm. Movie is rendered at 10 frames per seconds.

Movie S3: Fluorescence recovery after photobleaching of labeled PorH-ALFA. Cells (H3922, Δ*porH* P_{tac}*::porH-ALFA*) were prelabled with 30 µM 6-TMR-Tre (MM), 100 µM HADA (PG) and 12.5 nM Atto488-NB (PorH), spotted onto a 2% agarose pad, and imaged on a Nikon AX confocal microscope. Photobleaching was achieved by exposing cells to a 405nm, 488nm and 561nm laser at 100% power using 2 µs dwell time per pixel. Three examples of FRAP are shown, MM = mycomembrane, PG = peptidoglycan, scale bar = 2 µm. Movie is rendered at 7 frames per seconds.

Movie S4: Fluorescence recovery after photobleaching of ProtX. Cells (DB042*,* P_{tac}*::protX-ALFA*) were prelabeled with 30 µM 6-TMR-Tre (MM) and 12.5 nMAtto488-NB (PorH), spotted onto a 2% agarose pad, and imaged on a Nikon AX confocal microscope. Photobleaching was achieved by exposing cells to a 488nm and 561nm laser at 100% power using 2 µs dwell time per pixel. Three examples of FRAP are shown, MM = mycomembrane, scale bar = 2 µm. Movie is rendered at 6 frames per seconds.

Movie S5: Fluorescence dilution assay of PorH. Cells (H3922, Δ*porH* P_{tac}*::porH-ALFA*) were prelabled with 30 µM 6-TMR-Tre (MM), 100 µM HADA (PG) and 12.5 nM Atto488-NB (PorH), spotted onto a 2% agarose pad, and imaged over 4 hrs with a 20 min acquisition frame rate. Individual fluorescence channels are shown as merged overlays with the phase contrast channel. MM = mycomembrane, PG = peptidoglycan, scale bar = 3µm. Movie is rendered at 5 frames per seconds.

Movie S6: Fluorescence dilution assay of ProtX. Cells (H3922, Δ*porH* P_{tac}*::porH-ALFA*) were prelabled with 30µM 6-TMR-Tre (MM), 100µM HADA (PG) and 12.5 nM Atto488-NB (PorH), spotted onto a 2% agarose pad, and imaged over 4 hrs with a 20 min acquisition frame rate. Individual fluorescence channels are shown as merged overlays with phase contrast channel. MM = mycomembrane, PG = peptidoglycan, scale bar = 3 µm. Movie is rendered at 5 frames per seconds.

Table S1: Strain list

Strain number	Genotype	Strain construction	Source/reference
H60	MB001 (ATCC 13032 ΔCGP1 (cg1507-gp1524) ΔCGP2 (cg1746-1752) ΔCGP3 (cg1890-cg2071))		(6)
H241	*divIVA::divIVA-mScarlet*	Replacement of native *divIVA* with *divIVA-mScarlet* fusion	(7)
H1111	*ΔprotX (cgp_2875)*	Deletion of *protX* (allelic exchange)	(1)
H1177	MB001 pEMH25	H60/pEMH25	This study
H1241	*ΔporH (cgp_3009)*	Deletion of *porH* (allelic exchange)	(1)
H3249	*ΔporH* pEWL103	H1241/pEWL103	This study
H1248	*ΔporH* pEMH25	H1241/pEMH25	(1)
H1249	*ΔporH* pEMH26	H1241/pEMH26	(1)
H1448	*ΔporH* pEMH27	H1241/pEMH27	(1)
H3369	*ΔporH* pEMH27 pEWL103	H1448/pEWL103	This study
H3398	*ΔporH eccB3::kan* pEMH27	H3369	This study
H3399	*ΔporH eccC5::kan* pEMH27	H3369	This study
H3400	*ΔporH eccD2::kan* pEMH27	H3369	This study
H1616	*ΔporH* pEMH145	H1241/pEMH145	This study
H5052	*ΔporH attB2(Zeo)::EV*	H1241/pEMH691	This study
H5078	*ΔporH attB2(Zeo)::EV* pEMH27	H5052/pEMH27	This study
H5063	*ΔporH cmt1::kan attB2(Zeo)::EV*	H3521/pEMH691	This study
H5079	*ΔporH cmt1::kan attB2(Zeo)::EV* pEMH27	H5063/pEMH27	This study
H5060	*ΔporH cmt1::kan attB2(Zeo)::cmt1*	H3521/pEMH692	This study
H5080	*ΔporH cmt1::kan attB2(Zeo)::cmt1* pEMH27	H5060/pEMH27	This study
H3922	*ΔporH* pEMH600	H1241/pEMH644	This study
H1152	*ΔporA (cgp_3008)*	Deletion of *porA* (allelic exchange)	This study

H1247	Δ*porAH (cgp_3008-cgp_3009)*	Deletion of *porA/porH* (allelic exchange)	This study
H3542	Δ*porAH* pEWL103	H1247/pEWL103	This study
H1493	Δ*porAH* pEMH25	H1247/pEMH25	This study
H3019	Δ*porAH* pEMH27	H1247/pEMH27	This study
H3291	Δ*porAH* pEMH114	H1247/pEMH114	This study
H3292	Δ*porAH* pEMH115	H1247/pEMH115	This study
H3293	Δ*porAH* pEMH486	H1247/pEMH486	This study
H3294	Δ*porAH* pEMH487	H1247/pEMH487	This study
H3521	Δ*porH cmt1::kan*	Disruption of *cmt1* in H1241	This study
H3655	Δ*porH cmt1::kan* pEMH27	H3521/pEMH27	This study
H3763	Δ*porAH cmt1::kan*	Disruption of *cmt1* in H1247	This study
H3657	Δ*porAH cmt1::kan* pEMH27	H3763/pEMH27	This study
H3765	Δ*porAH cmt1::kan* pEMH114	H3763/pEMH114	This study
DB016	*divIVA::divIVA-mScarlet* pEMH600	H241/pEMH600	This study
DB042	MB001 pDB005	H60/pDB005	This study
DB044	Δ*protX* pDB006	H1111/pDB006	This study
DB045	Δ*protX* pDB005	H1111/pDB005	This study

Table S2: Plasmids used in this study

Plasmid name	Information	Source
pEWL89	pCRD206(Apra^R)::P$_{sod}$-*cre*	(1)
pEWL103	pCRD206(Apra^R)::P$_{tac}$ riboE1-SSAP/SSB	(1)
pCRD206	Kan^R*, sacB* counterselection, temperature-sensitive origin	(8)
pEMH10	pCRD206::*porA*	This study
pEMH16	pCRD206::*porAH*	This study
pEMH25	P$_{sod}$ empty vector (Cam^R, pGA1 mini replicon, constitutive expression, native 6x His deleted)	(1)
pEMH117	Deletion of native 6x His to match pEMH25	(1)
pEMH26	pEMH25::*porH*	(1)
pEMH27	pEMH25::*porH-His*	(1)
pEMH87	pEMH25::*porH$_{S56A}$-His*	This study
pEMH145	pEMH25::*porH*-ALFA	This study
pEMH114	pEMH25::*porH-His/porA*	This study
pEMH115	pEMH25::*porH$_{S56A}$-His/porA*	This study
pEMH486	pEMH25::*porH-His/porA$_{S15A}$*	This study
pEMH487	pEMH25::*porH$_{S56A}$-His/porA$_{S15A}$*	This study
pEMH120	P$_{tac}$ empty vector (pACM246-derived), *eGFP* deleted, insertion of BamHI site in MCS	(1)
pEMH604	pEMH120::*porH*	This study
pEMH600	pEMH120::*porH-ALFA*	This study
pACM185	P$_{sod}$ riboE1 empty vector (Kan^R, pK-PIM derivative, theophylline inducible)	(9)
pACM64	P$_{sod}$ riboE1(Kan^R)::*cmt1*	(9)
pEMH691	P$_{sod}$ riboE1 empty vector (Zeo^R, pK-PIM derivative, theophylline inducible)	This study
pEMH692	P$_{sod}$ riboE1(Zeo^R)::*cmt1*	This study
pDB005	pEMH120::*protX-ALFA*	This study
pDB006	pEMH120::*protX*	This study

Table S3: Oligonucleotides used in this study

Primer name	Sequence (5' → 3')	Description (associated plasmid)
BH288	ggataacttcgcatcctaatgacctaggtg	Forward, to make S56A mutation in *porH* by site-directed mutagenesis (pEMH87)
BH256	agattctcgccggtggtg	Reverse, to make S56A mutation in *porH* by site-directed mutagenesis (pEMH87)
BH562	ctgcgccgccgcctgaccgaatgacctaggtgc ctggcg	Forward, to insert C-terminal ALFA tag in *porH* (pEMH145)
BH563	ttcttcttccaggcggctcggggaagagaagttatc cagattctcgc	Reverse, to insert C-terminal ALFA tag in *porH* (pEMH145)
BH421	ctaggtgcctggcggcag	Forward, to linearize pEMH27/pEMH87 vectors (pEMH114)
BH422	gtcagtggtggtggtggtg	Reverse, to linearize pEMH27/pEMH87 vectors (pEMH114)
BH423	ccaccaccaccaccactgacgagaaatccga tttggctg	Forward, to amplify *porA* and *porH/porA* intergenic region from chromosome (pEMH114)
BH424	tactgccgccaggcacctagttagccaagcag accgatg	Reverse, to amplify *porA* and *porH/porA* intergenic region from chromosome (pEMH114)
BH257	tgatgtccttgcaggctccggcc	Forward, to mutate residue S15A in *porA* by site-directed mutagenesis (pEMH486/pEMH487)
BH258	aggtttccaaggaactcgtaaac	Reverse, to mutate residue S15A in *porA* by site-directed mutagenesis (pEMH486/pEMH487)
BH1570	gcgaaaggatttttacatgatggatctttccc ttctcaagg	Forward, to amplify *porH-ALFA* for insertion into pEMH120 digested with AvrII/BamHI (pEMH600)
BH1572	gcgctactgccgccaggcactcattcggtcag gcggcg	Reverse, to amplify *porH-ALFA* for insertion into pEMH120 digested with AvrII/BamHI (pEMH600)
BH171	tcagaattggttaaaaaggatctagg	Forward, to linearize pK-PIM derivative vectors (pEMH691/pEMH692)
BH172	cgcacagatgcgtaaggag	Reverse, to linearize pK-PIM derivative vectors (pEMH691/pEMH692)
BH173	tctccttacgcatctgtgcggtacctctatctggt gccctaaac	Forward, to amplify zeocin-resistance cassette for insertion into pK-PIM derivative vectors (pEMH691/pEMH692)
BH174	tcctttttaaccaattctgatcagtcctgctcctc ggc	Reverse, to amplify zeocin-resistance cassette for insertion into pK-PIM derivative vectors (pEMH691/pEMH692)
BH1	agtcgacctgcaggcatg	Forward, to linearize pCRD206 (pEMH10)
BH2	atccaacagggacaccag	Reverse, to linearize pCRD206 (pEMH10)
BH7	gcagaataaataaatcctggtgtccc	Forward, diagnostic primer for pCRD206 derived vectors
BH8	gggtaacgccagggttttcc	Reverse, diagnostic primer for pCRD206 derived vectors

BH95	tcctggtgtccctgttggatacctcaattgccct cccg	Forward, to amplify upstream region of *porA* (pEMH10)
BH96	gcagaccgatgttttccattttaaattctcct attaagagttgag	Reverse, to amplify upstream region of *porA* (pEMH10)
BH25	aatggaaaacatcggtctgcttggctaa ttaac	Forward, to amplify downstream region of *porA* (pEMH10)
BH26	tgcatgcctgcaggtcgactggcggatagcaaaa cggg	Reverse, to amplify downstream region of *porA* (pEMH10)
BH45	tcctggtgtccctgttggatggcgagtacgtcg acctc	Forward, to amplify upstream region of *porAH* (pEMH16)
BH132	gcagaccgataagatccatgagaaatct ccttgag	Reverse, to amplify upstream region of *porAH* (pEMH16)
BH133	catggatcttatcggtctgcttggctaattaac	Reverse, to amplify downstream region of *porAH,* used with primer BH26 (pEMH16)
BH65	taacatttctgcaggtcaag	Forward, diagnostic primer for *porH*
BH66	tcagcaactgcgcca	Reverse, diagnostic primer for *porH*
BH67	ccgctcttcagagcatcc	Forward, diagnostic primer for *porA*
BH68	ggttccatctggacgcaga	Reverse, diagnostic primer for *porA*
BH71	tgcggtatttcacaccgcata	Forward, diagnostic primer for pEMH25 derivatives
BH250	ccggcggatttgtcctactca	Reverse, diagnostic primer for pEMH25 derivatives
BH1458	aggtcagggctaccaaccacaagttca cgagggaaagacgtatgaagctttccgtga tggtaacttcacg	Forward, recombineering primer for *cmt1*
BH1459	aaaggcagccggtttcaatgttaaatct gtttctaggcctctagctcaaaagccgtca attgtctgattc	Reverse, recombineering primer for *cmt1*
BH81	cagagatttttggctcgtta	Forward, diagnostic primer for *cmt1*
BH82	gtgactgtcgcagcaag	Reverse, diagnostic primer for *cmt1*
BH1304	cctggattaggggatgtaaagatctaaagctgggggaat acatggctgaaggctccgtgatggtaacttcacg	Forward, recombineering primer for *eccB3* (*cgp_0661*)
BH1305	caccatactccttaataggtgggcgcgagcgctgtctcttt tgctaattcactagccgtcaattgtctgattc	Reverse, recombineering primer for *eccB3* (*cgp_0661*)
BH1306	ATGAGCAGGCCATCGGTGT	Forward, diagnostic primer for *eccB3*
BH1307	CGGATCAATGGCAATCACTC	Reverse, diagnostic primer for *eccB3*

BH1308	CATATAGCACCGGCTTAACAGGCCGGTGCTATTCTGTTCGCATGACTTCGtccgtgatggtaacttcacg	Forward, recombineering primer for *eccC5* (*cgp_2498*)
BH1309	GGGCGCTGAGCGTCTACCATTGCATGGTCCTTTTAGCTGAAAGTGAGGAAagccgtcaattgtctgattc	Reverse, recombineering primer for *eccC5* (*cgp_2498*)
BH1310	TCTGGAACGTTGATTACGAC	Forward, diagnostic primer for *eccC5*
BH1311	TAGTGAATACGTCCGGCGC	Reverse, diagnostic primer for *eccC5*
BH1312	ggactcaccgcaggtggcgtgaaagcctagactagatactcatgagtgcttccgtgatggtaacttcacg	Forward, recombineering primer for *eccD2* (*cgp_0830*)
BH1313	tctggaattatcgtggcgttggcgcaggaactttaattagggcggcgcttagccgtcaattgtctgattc	Reverse, recombineering primer for *eccD2* (*cgp_0830*)
BH1314	cttccgcatgatcctgctt	Forward, diagnostic primer for *eccD2*
BH1315	tgctgatcggaccgttggt	Reverse, diagnostic primer for *eccD2*
DB007	gtgcctggcggcagtag	Forward, to linearize pEMH120 vectors
DB008	catgtaaaaaatcctttcgctagcaaattg	Reverse, to linearize pEMH120 vectors
DB016	gatgcctggcagttccctac	Forward, diagnostic primer for insertions into pEMH120
DB017	cgcactcccgttctggataatg	Reverse, diagnostic primer for insertions into pEMH120
DB040	ccgagccgcctggaagaagaactgcgccgccgcctgaccgaatgagtgcctggcggcagtag	Forward, to incorporate ALFA into linearized pEMH120 vectors
DB041	gagcggataacaatttgctagcgaaaggattttttacatgatgacctctgtattcgatatcatc	Forward, to amplify ProtX for insertion into pEMH120 linearized with DB017 and DB040 (pDB005)
DB042	tcattcggtcaggcggcggcgcagttcttcttccaggcggctcggggaagaagcggtaacgatg	Reverse, to amplify ProtX-ALFA for insertion into pEMH120 linearized with DB017 and DB040 (pDB005)
DB070	tcaggtgggaccaccgcgctactgccgccaggcacctaggaagaagcggtaacgatg	Reverse, to amplify ProtX for insertion into pEMH120 linearized with DB016 and DB017 (pDB006)

REFERENCES

1. Hart EM, Lyerly E, Bernhardt TG. 2024. The conserved σ^D envelope stress response monitors multiple aspects of envelope integrity in corynebacteria. *PLoS Genet* 20:e1011127. PMID: 38829907.
2. Ravasi P, Peiru S, Gramajo H, Menzella HG. 2012. Design and testing of a synthetic biology framework for genetic engineering of *Corynebacterium glutamicum. Microb Cell Fact* 11:147. PMID: 23134565.
3. Seeliger JC, Topp S, Sogi KM, Previti ML, Gallivan JP, Bertozzi CR. 2012. A Riboswitch-Based Inducible Gene Expression System for Mycobacteria. *PLoS ONE* 7:e29266. PMID: 22279533.
4. Oram M, Woolston JE, Jacobson AD, Holmes RK, Oram DM. 2007. Bacteriophage-based Vectors for Site-specific Insertion of DNA in the Chromosome of Corynebacteria. Gene 391:53–62. PMID: 17275217.
5. Jumper J, Evans R, Pritzel A, Green T, Figurnov M, Ronneberger O, Tunyasuvunakool K, Bates R, Žídek A, Potapenko A, Bridgland A, Meyer C, Kohl SAA, Ballard AJ, Cowie A, Romera-Paredes B, Nikolov S, Jain R, Adler J, Back T, Petersen S, Reiman D, Clancy E, Zielinski M, Steinegger M, Pacholska M, Berghammer T, Bodenstein S, Silver D, Vinyals O, Senior AW, Kavukcuoglu K, Kohli P, Hassabis D. 2021. Highly accurate protein structure prediction with AlphaFold. *Nature* 596:583–589. PMID: 34265844.
6. Baumgart M, Unthan S, Rückert C, Sivalingam J, Grünberger A, Kalinowski J, Bott M, Noack S, Frunzke J. 2013. Construction of a prophage-free variant of *Corynebacterium glutamicum* ATCC 13032 for use as a platform strain for basic research and industrial biotechnology. *Appl Environ Microbiol* 79:6006–6015. PMID: 23892752.
7. Lim HC, Sher JW, Rodriguez-Rivera FP, Fumeaux C, Bertozzi CR, Bernhardt TG. 2019. Identification of new components of the RipC-FtsEX cell separation pathway of Corynebacterineae. *PLoS Genet* 15:e1008284-31. PMID: 31437147.
8. Okibe N, Suzuki N, Inui M, Yukawa H. 2011. Efficient markerless gene replacement in *Corynebacterium glutamicum* using a new temperature-sensitive plasmid. *J Microbiol Methods* 85:155–163. PMID: 21362445.
9. McKitterick AC, Bernhardt TG. 2022. Phage resistance profiling identifies new genes required for biogenesis and modification of the corynebacterial cell envelope. *Elife* 11:e79981. PMID: 36350124.
